# Are threatened species special? An assessment of Dutch bees in relation to land use and climate

**DOI:** 10.1002/ece3.10326

**Published:** 2023-07-26

**Authors:** Merijn Moens, Jacobus C. Biesmeijer, Saskia G. T. Klumpers, Leon Marshall

**Affiliations:** ^1^ Naturalis Biodiversity Center Leiden The Netherlands; ^2^ Institute of Environmental Sciences (CML) Leiden University Leiden The Netherlands; ^3^ Agroecology Lab, Interfaculty School of Bioengineering Université libre de Bruxelles (ULB) Brussels Belgium

**Keywords:** bees, climate, land use, red list, species distribution models, threatened species

## Abstract

Red Lists are widely used as an indicator of the status and trends of biodiversity and are often used in directing conservation efforts. However, it is unclear whether species with a Least Concern status share a common relationship to environmental correlates compared to species that are on the Red List. To assess this, we focus here on the contribution and correlates of land use, climate, and soil to the occurrence of wild bees in the Netherlands. We used observation data and species distribution models to explain the relation between wild bees and the environment. Non‐threatened bees had a relatively higher variable importance of the land use variables to their models, as opposed to the climate variables for the threatened bees. The threatened bees had a smaller extent of occurrence and occupied areas with more extreme climatic conditions. Bees with a Least Concern status showed more positive responses to urban green spaces and Red List species showed a different response to climatic variables, such as temperature and precipitation. Even though Red List bees were found in areas with a higher cover of natural areas, they showed a more selective response to natural land use types. Pastures and crops were the main contributing land use variables and showed almost exclusively a negative correlation with the distribution of all wild bees. This knowledge supports the implementation of appropriate, species‐specific conservation measures, including the preservation of natural areas, and the improvement of land use practices in agricultural and urban areas, which may help mitigate the negative impacts of future global change on species' distributions.

## Graphical abstract

We found that land use contributes relatively more to the distribution of non‐threatened species and climate is a relatively more important determinant for the distribution of threatened species. Additionally, threatened species showed distinct responses to land use categories compared to non‐threatened species: more selective response to natural land uses, and less positive response to both urban land uses and habitat heterogeneity (number of land use categories).

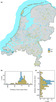

## 1 INTRODUCTION

Recent biodiversity loss has led to the assertion that the world is amid a sixth mass extinction event (Ceballos et al., 2015). Among all invertebrates, vertebrates, and plants, an average of 25% of species is at risk of extinction (categorized by the IUCN as vulnerable, endangered, or critically endangered; IPBES, 2019). The IUCN Red List is an important tool for raising awareness, increasing scientific knowledge, and for bringing together relevant stakeholders for the conservation of threatened species (Betts et al., 2020). In five decades, it has developed from a small subjective list of threatened species to an extensive list with quantitative criteria for the extinction risk status across taxonomic groups (Betts et al., 2020).

The IUCN Red List assessments use distribution and abundance data to determine trends in extent and area of occupancy (IUCN, 2016). They do not involve modeling and do not incorporate the role of environmental variables on distribution patterns (IUCN, 2016; Natural, 2001; Syfert et al., 2014). Species distribution models (SDMs) are models that can be used to estimate species niches and distributions in unsampled areas (Elith & Leathwick, 2009).The SDMs can be used to predict the distribution of a species in a certain time period and geographic region, but they can also be used to statistically estimate the relationship between environment and species (Araújo et al., 2019). The SDMs are rarely used in Red List assessments (Syfert et al., 2014), even though they can provide insight in the correlations between environment and species occurrences and thus explain differences in the ecology of species with a Least Concern status (LC), referred to as non‐threatened species in this study, and species with a Red List status (RL) with any risk status besides LC, referred to as threatened species. Lack of data and limited uptake of methods are important obstacles in the long‐term growth and consistency of the RLs and models can aid in providing standardized estimates for the assessment (reviewed in Cazalis et al., 2022).

Climatic variables and land use variables are widely used in SDMs (Booth, 2018; Cordonnier et al., 2019; Dorrough & Scroggie, 2008; Marshall et al., 2021; Santos et al., 2021) and climate and land use are among the most important drivers of change to biodiversity (IPBES, 2019). In general, studies have not shown a consistent stronger effect of either climate change or land use and land cover change on the distribution of species among different taxonomic groups (Santos et al., 2021).The importance of land use and climate on species distributions may change over time (Aguirre‐Gutiérrez et al., 2017), scale (Martin et al., 2013), and thematic resolution (Marshall et al., 2021). Land use can influence the distribution of species through mechanisms such as habitat fragmentation (Püttker et al., 2020), agricultural land expansion (Dorrough & Scroggie, 2008), and urbanization (Cordonnier et al., 2019). Global patterns of land use are often correlated with climate (Dale, 1997; Thuiller et al., 2004). However, land use and climate act on different scales (Martin et al., 2013; Santos et al., 2021) and climate has a stronger effect on population distribution and land use on dispersal, population viability, and reproductive output (Santos et al., 2021).

Differences in the environmental requirements of threatened and non‐threatened species have been found in several groups of species. For example, threatened plants tend to require a more restricted range of soil pH values compared to the LC plants (Gustafsson, 1994; Pärtel et al., 2004). Besides environmental requirements, non‐threatened and threatened species tend to differ in their biological traits. For example, threatened seabirds tend to be larger than non‐threatened seabirds (Gaston & Blackburn, 1995; Richards et al., 2021) and threatened seabirds were found to occupy a smaller habitat breadth compared to non‐threatened species (Richards et al., 2021). Red Listed bees were found to be larger than non‐threatened bees, and they visited more threatened food plants as a pollen source (Scheper et al., 2014). Threatened species tend to have a lower species richness of parasites compared to non‐threatened species in primates (Altizer et al., 2007) and plants (Gibson et al., 2010). Moreover, plants with a higher floral complexity are more likely to be threatened than plants with simpler flowers (Stefanaki et al., 2015).

**FIGURE 1:**
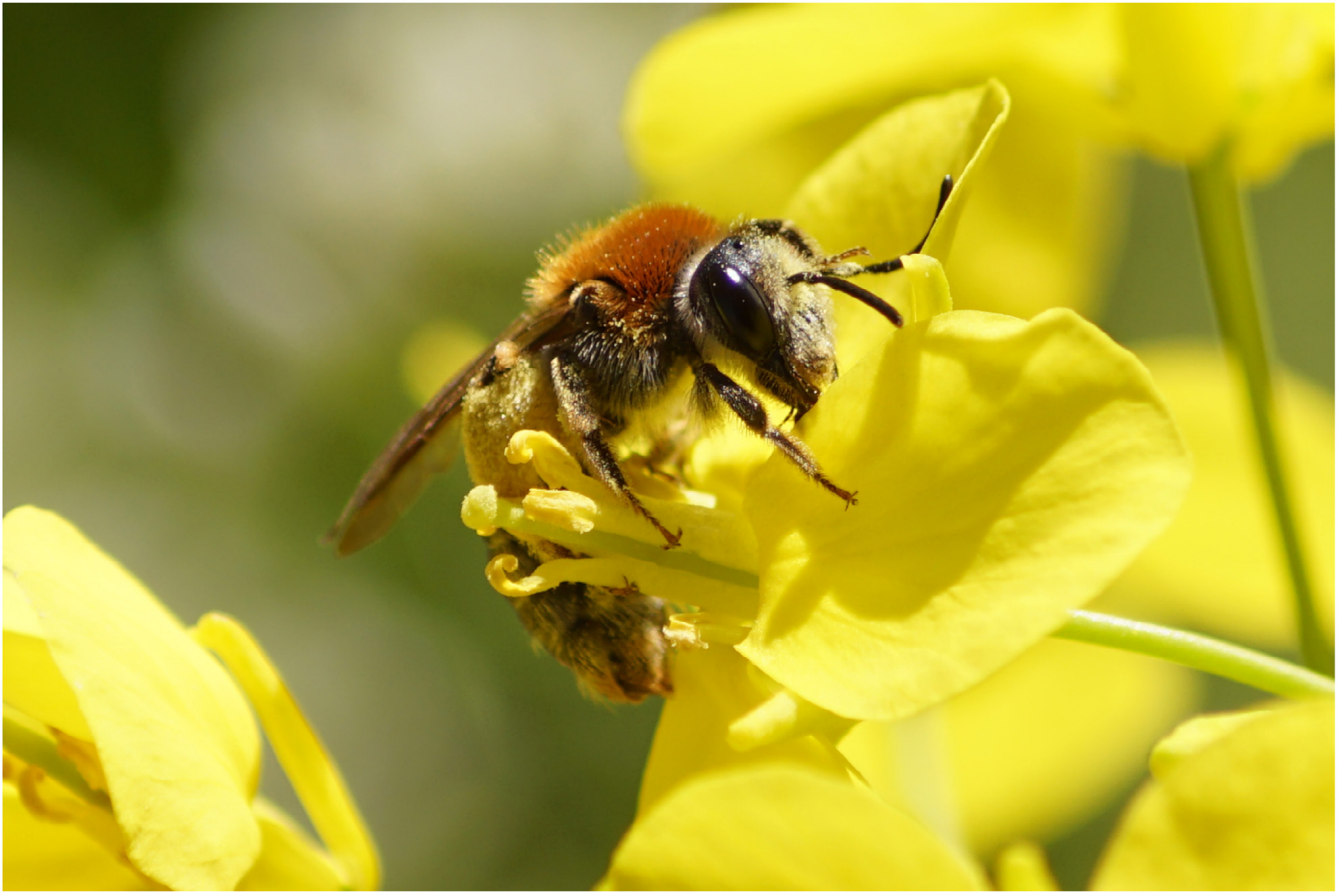
*Andrena haemorrhoa* represents a common species in the Netherlands and is classified as Least Concern on the Dutch wild bee Red List.

There is a lack of knowledge on factors determining the distribution of insects and even though SDMs are applied to a wide range of species, insects are an underrepresented group (Lobo, 2016). Bees in the Netherlands are a suitable group of organisms to study differences between threatened and non‐threatened species as there are over 300 bee species, of which 181 (55%) are listed as threatened and 150 as non‐threatened (Reemer, 2018; Figure 1). The Dutch Red List is based on both the trend in the distribution or number of reproductive individuals since 1950 and the actual distribution (Reemer, 2018), while for the European Red List 79% of the bee species do not have sufficient data for population trends (Nieto et al., 2014). As the Netherlands has a small land surface area (33,647 km^2^; CBS, 2022) and we are using fine resolution spatial data, we are expecting to find a high contribution of land use as opposed to a higher contribution of climate at a larger scale. Similar to the rest of Europe, the Netherlands has been affected by agricultural intensification, one of the most influential drivers of biodiversity decline in Europe (Henle et al., 2008). In the last century, there has been an increase in the agricultural intensification, for example, massive application of fertilizers, in the Netherlands, and the proportion of semi‐natural habitat for pollinators has decreased to only one‐fifth of its original area in the Netherlands since 1900 (Scheper et al., 2014). Besides land use and climate, soil characteristics are an important factor for many wild bees (reviewed in Antoine & Forrest, 2021) and around 250 of the bees in the Netherlands are ground‐nesting (Peeters et al., 2012). Here we evaluate whether LC and RL bees differ in their location and distribution among land use, climate, and soil categories. Following this, we compare the response of LC and RL bees to specific types of natural, urban, and agricultural land uses.

## 2 MATERIALS AND METHODS

### 2.1 Abiotic data acquisition and processing

As explanatory variables for the SDMs, we used Dutch national climate, land use, and soil data at a spatial resolution of 100 m × 100 m. For all modeled species, the study extent included the land within the political boundary of the Netherlands, excluding the grid cells with complete cover of saltwater. We selected uncorrelated land use variables from various sources that can be classified as natural (Inter Provinciaal Overleg, 2016), urban (CBS, 2012) and agricultural areas (Ministerie van Economische Zaken, 2015). The values of the variables are the percentage cover of the respective land use types within the grid cells. We calculated an additional variable, the summed number of land uses, which is the sum of land uses categories per grid cell. As climate variables, we used daily temperature and rainfall data obtained from the Royal Netherlands Meteorological Institute from 2005 to 2014. We aggregated the climate data to bioclimatic variables (Fick & Hijmans, 2017) that represent ecologically relevant variables using the dismo package version 1.3‐3 (Hijmans et al., 2017). Of these bioclimatic variables, we selected a set of uncorrelated variables that included annual mean temperature (bio 1), temperature seasonality (bio 4), mean daily mean air temperatures of the wettest quarter (bio 8), annual precipitation amount (bio 12), precipitation amount of the driest month (bio 14), and precipitation seasonality (bio 15; Fick & Hijmans, 2017). The Pearson's correlation coefficient was determined in the usdm package version 1.1.18 (Naimi et al., 2014) with a threshold at 0.7, commonly used as a cut‐off for collinearity (Dormann et al., 2013). We used data from the Dutch soil map from 2006 (Grondsoortenkaart, 2006) that included the main soil types in the Netherlands: sand, peat, light clay, heavy clay, light zavel, heavy zavel, loam, and moerig op zand (Silvis & Voskuilen, 2016) described in Table S1.

### 2.2 Species data acquisition and cleaning

**FIGURE 2:**
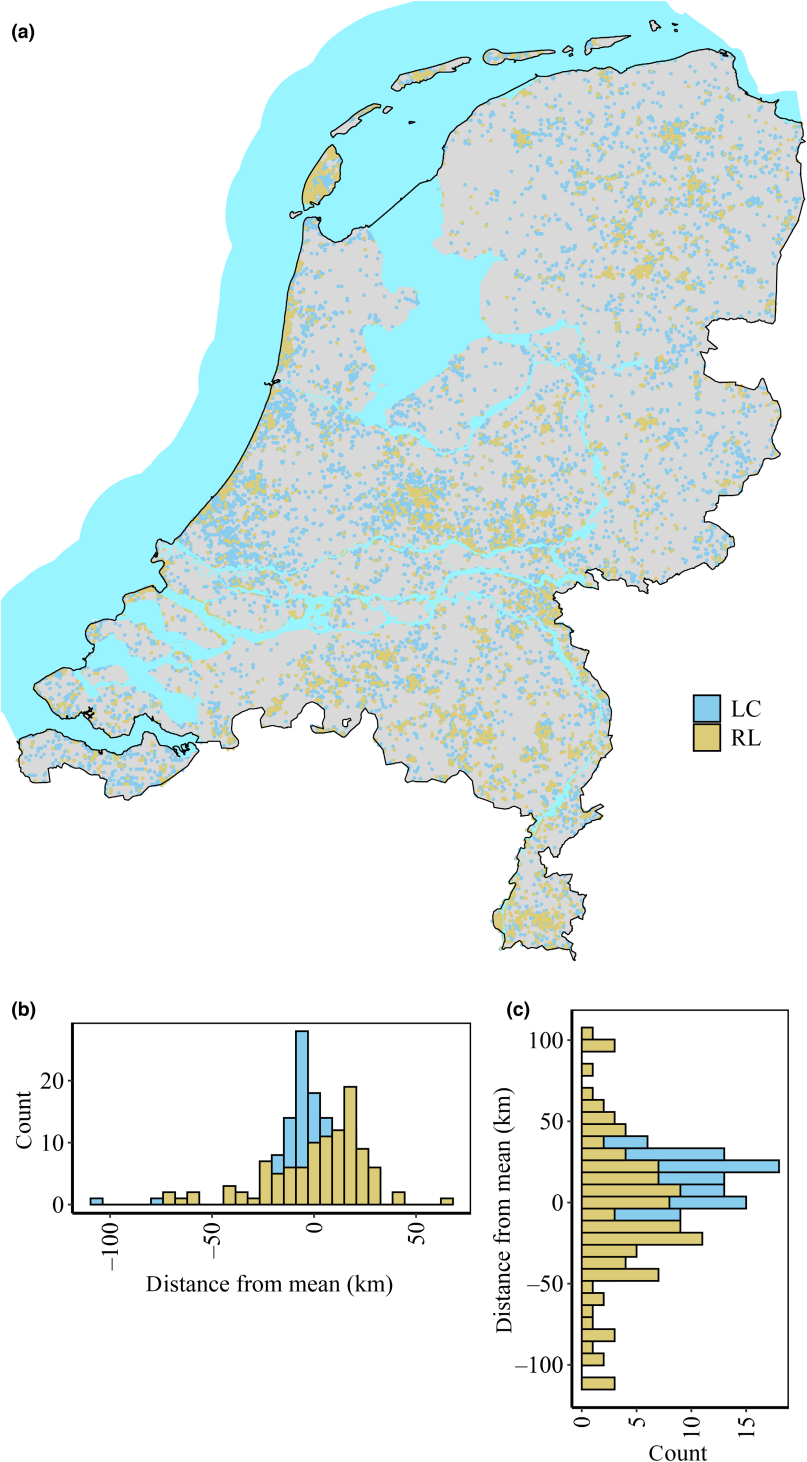
The spatial distribution of observation data for Red List and Least Concern wild bee species in the Netherlands (a) and their difference in spatial distribution on the longitudinal (b) and latitudinal (c) axis. (b, c) The distance in kilometers between the average longitude and latitude orientation of the modeled species and the average longitude and latitude values of all observations.

The bee observation data originated from the European Invertebrate Survey Netherlands (EIS, 2020). The data includes observations from various sources in the period from 2004 to 2019, and it is collected by both professionals and amateurs. The observations were filtered using spatial thinning at 300 m in the spThin package version 0.2.0 (Aiello‐Lammens et al., 2015). Only the bee species with at least 15 observations were modeled, resulting in a total of 222 bee species (Table S2), across the Netherlands (Figure 2). We used the Dutch Red List (Reemer, 2018) and European Red List (Nieto et al., 2014) for dividing the bees into non‐threatened (LC) and threatened (RL) bees (Syfert et al., 2014).

### 2.3 Model building and evaluation

Species distribution models represent tools that can numerically combine environmental data and species observations or abundance (Elith & Leathwick, 2009). MaxEnt is a machine learning algorithm for species distribution modeling that includes controls for model complexity (Phillips et al., 2006). It performs solidly among other presence‐only algorithms (Kaky et al., 2020; Lissovsky & Dudov, 2021), especially when the sample size is smaller (Aguirre‐Gutiérrez et al., 2013). Additionally, the MaxEnt algorithm is more efficient in handling collinearity among predictor variables (De Marco & Nóbrega, 2018; Feng et al., 2019). For modeling bee distributions, we used MaxEnt version 3.4.1 (Phillips et al., 2006) in R (R Core Team, 2020) with the packages: dismo version 1.3‐9 (Hijmans et al., 2017) and ENMeval version 2.0.4 (Muscarella et al., 2014). We ran a range of regularization multiplier values and feature classes, including linear (L), quadratic (Q), hinge (H), threshold (T), and product (P) features, which represent functions of the predictor variables and can add potential complexity (Phillips & Dudík, 2008). The regularization multiplier, which controls allowable model complexity, ranged from 1 to 15, where higher values increasingly penalize complexity (Phillips & Dudík, 2008). We tested the following combinations of feature types: L, L + Q, H, L + Q + H, L + Q + H + P, L + Q + H + P + T (Data S1). From these models, the model with the lowest AICc (Burnham & Anderson, 2002) was selected per individual modeled species and ties were broken with lowest 10% training omission rate. Other evaluation measures that have been calculated, include the average continuous Boyce Index (Hirzel et al., 2006) and Minimum Training Presence omission rate (Muscarella et al., 2014), the area under the curve (AUC) of the receiver operating characteristic for SDMs of the calibration and evaluation dataset (Radosavljevic & Anderson, 2014) and they can be found in Data S2. The evaluation measures were calculated based on the evaluation dataset and the division between calibration and evaluation data was done following the procedure of a spatial block validation (Muscarella et al., 2014) with four distinct geographical areas: one part evaluation data and three parts calibration data. The number of background points was set to 10,000 from the whole study region. A summary of the models following the ODMAP protocol (Zurell et al., 2020) can be found in Table S3.

### 2.4 Statistical analyses

Differences in the extent of occurrence, location, and percentage of natural land cover (sum of the percentage cover of individual natural land use variables) between LC and RL were compared using a Wilcoxon signed‐rank test (Hollander et al., 2013). Additionally, we wanted to see whether RL species were found in more extreme climate conditions compared to LC species. We tested this by (i) first calculating the mean value of each bioclimatic variable across the observation range of each modeled species, (ii) for each species we then calculated how many standard deviations this average was away from the average value of that variable across the whole study extent, and (iii) finally, we statistically tested whether there was a significant difference between the mean number of standard deviations for LC and RL species using a Kruskal–Wallis test (Kruskal & Wallis, 1952) with a post‐hoc Dunn's test (Dunn, 1964).

Permutation importance is a measure of variable importance in the SDMs, and it is calculated by randomizing the values of a variable for presence and background samples while keeping the other variables constant (Jarnevich et al., 2016; Kalle et al., 2013). It is expressed as the change in training AUC of the final model converted to percentages (Kalle et al., 2013). Differences in permutation importance from the SDMs of the land use, climate, and soil variables, summed per category, were compared between the best performing models of the LC and RL bees with base R and the FSA package version 0.9.4 (Ogle et al., 2023), using a Kruskal–Wallis test (Kruskal & Wallis, 1952) with a post‐hoc Dunn's test (Dunn, 1964). We adjusted the *p*‐values for multiple comparisons with the Holm method (Holm, 1979). Using a linear regression and a nonparametric rank‐based regression model in the Rfit package version 0.24.2 (Kloke & McKean, 2012), we calculated the relation between the variable importance of land use, climate, and soil and the RL status and extent of occurrence. The overall significance of the linear models has the null‐hypothesis that all of the coefficients are zero, and this was tested with the *F*‐statistic in base R (James et al., 2013). The best performing regression model was selected based on the lowest AICc values. The extent of occurrence is an often used measure in RL assessments (Syfert et al., 2014). It is the area that includes the outermost locations or inferred locations (Syfert et al., 2014) and it was calculated using the sf package version 1.0.9 (Pebesma, 2018).

The relationship between habitat suitability of the best performing model and each environmental variable was calculated using the response function in the dismo package version 1.3‐9 (Hijmans et al., 2017), resulting in marginal response curves with the other variables at their median value. The response of the species to each environmental variable was deemed positive or negative when the correlation coefficient (Spearman's) between the habitat suitability and the individual environmental variable in the marginal response curves was higher than .7, similar to (Marshall et al., 2021). If the variable was not included in the best performing SDM or when the habitat suitability and the individual environmental variable did not show a correlation coefficient (Spearman's) higher than .7, we considered the relation between the two as no response. The differences in responses of modeled species to variables were assessed with a Cochran–Mantel–Haenszel test (CMH; Cochran, 1954), using the post‐hoc test with the Fisher's exact test (Freeman & Halton, 1951), in the Rcompanion package version 2.4.1 (Mangiafico, 2021).

## 3 RESULTS

### 3.1 Differences in the observation data

**FIGURE 3:**
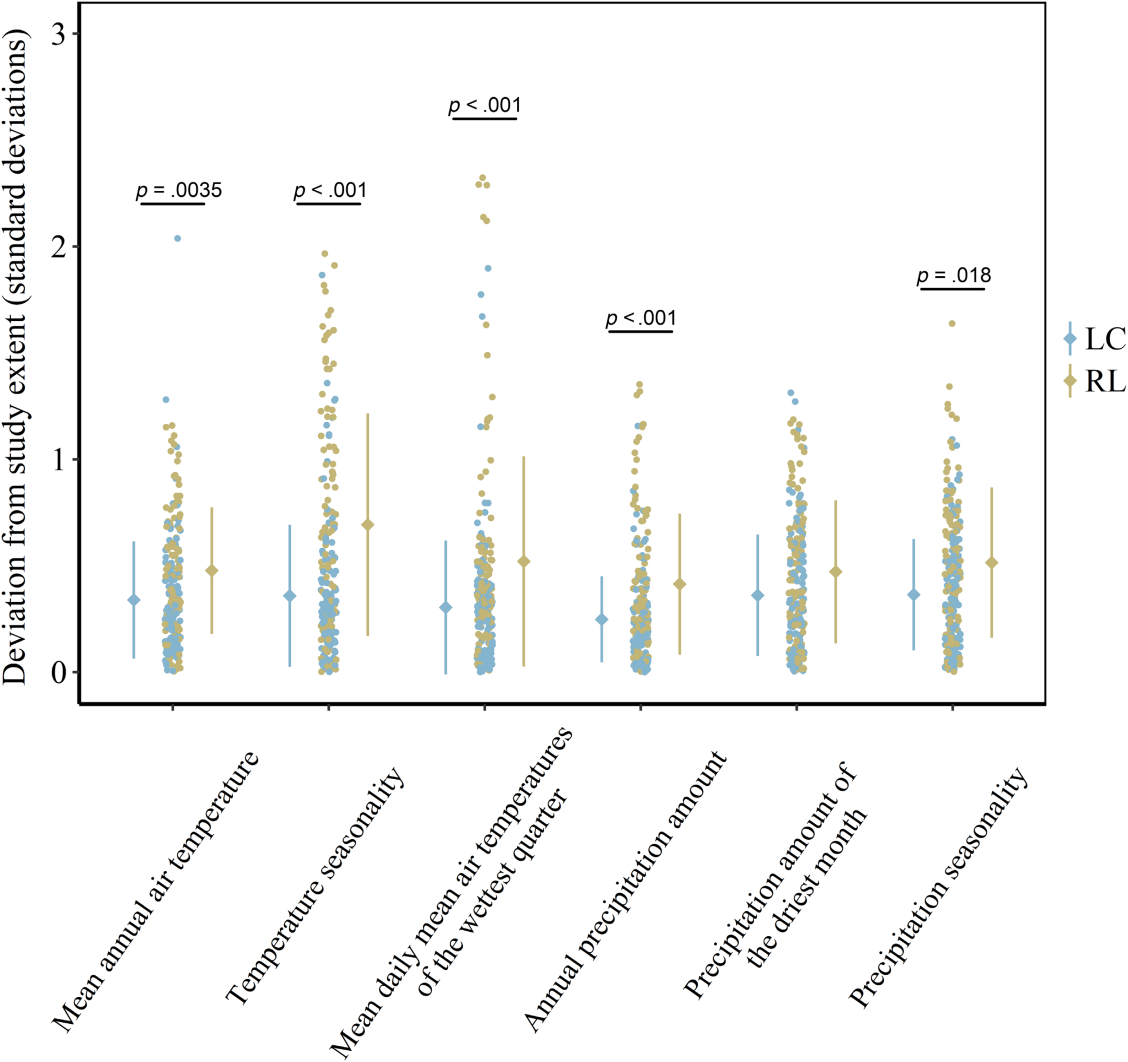
The graph shows the deviation, expressed as the standard deviation from the average of the bioclimatic variable in the Netherlands for Least Concern species (blue) and Red List species (red). The average of the values per species are based on the location of the observation data from the European Invertebrate Survey Netherlands (EIS, 2020). Significance values indicate the *p*‐value of the post‐hoc Dunn's test (Dunn, 1964) after a Kruskal–Wallis test (Kruskal & Wallis, 1952).

The extent of occurrence, location, percentage of natural land cover, and the values of the climate variables for the observations differed between LC and RL species. The LC wild bees differed significantly in their extent of occurrence from the RL bees (*W* = 10,135; *p* < .001), having an average of 40,468 (±993.42) km^2^ compared to an average of 25,675 (±1278.21) km^2^. The location of the LC bees' observations, averaged per species, were on average 3.38 km more toward the West of the Netherlands (*W* = 5010; *p* = .018) than the RL species and 12.07 km more to the North (*W* = 7422, *p* = .0074; Figure 2). Additionally, LC species were observed in areas with a lower percentage cover of natural areas (42.71% ± 1.31%) compared to the RL species (47.92% ± 1.90%) and this difference was significant (*W* = 5076, *p*‐value = .026). The observations of the LC species were closer to the mean of possible climatic values in the Netherlands compared to the RL species for bioclim 1 (Kruskal–Wallis chi‐squared [*χ* ^2^] = 114.44; *p* = .0035), bioclim 4 (*W* = 3058; *p* < .001), bioclim 8 (*p* < .001), bioclim 12 (*p* < .001), and bioclim 15 (*p* < .001; Figure 3).

### 3.2 Differences in the species distribution models

**FIGURE 4:**
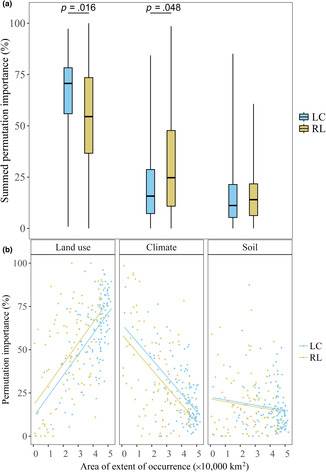
Difference in variable contribution of land use, climate, and soil to the distribution of Least Concern (LC) and Red List (RL) Dutch bees expressed as the summed permutation importance (a) and the relation between the summed permutation importance, RL status and the extent of occurrence of the species (b). The *R* ^2^ is calculated from a linear regression model, using the extent of occurrence and the RL status as explanatory variables.

The 117 LC species and 105 RL species differed in the summed permutation importance of land use, climate, and soil, and the extent of occurrence of the modeled species showed a strong correlation with the summed permutation importance. We found that land use variable importance was significantly more important for LC bees compared to RL bees using the summed permutation importance (59.71% ± 2.00% and 48.54% ± 2.58%, respectively; Kruskal–Wallis chi‐squared [*χ* ^2^] = 235; post‐hoc Dunn's test *p* = .016) (Figure 4a). The summed permutation importance of climate was significantly less important for the LC species than for the RL species (23.94% ± 1.68% and 32.85% ± 2.46%, respectively; *χ* ^2^ = 235; *p* = .048). The variable importance of soil variables did not differ significantly between LC and RL species (16.35% ± 1.26% and 17.65% ± 1.56%; *χ* ^2^ = 235; *p* = .83). We ran parametric and nonparametric models for the variable importance of land use climate and soil, since not all residuals had a normal distribution. The extent of occurrence was part of the regression model as an explanatory variable and the *p*‐values for parametric (*p*) and nonparametric (*p*_np_) were calculated. The best performing regression model for the land use variable importance, included a positive correlation of the extent of occurrence (*β*_1_; *p* < .001/*p*_np_ < .001) and the RL status (*β*_2_; *p* = .033/*p*_np_ = .037), and the model was significant (*p* < .001/*p*_np_ < .001; *R* ^2^ = .38/*R*_np_^2^ = .34; Figure 4b; Table S4). For the climate models the best performing model also resulted in the formula with a positive correlation with the extent of occurrence (*β*_1_; *p* < .001/*p*_np_ < .001) and RL status (*β*_2_; *p* = .038/*p*_np_ = .004), and the model was significant (*p* < .001/*p*_np_ < .001; *R* ^2^ = .33/*R*_np_^2^ = .29; Table S5). The best performing model for the variable importance of soil was the model with only a positive correlation with the extent of occurrence, but this model was not significant (*p* = .059/*p*_np_ = .039).

**FIGURE 5:**
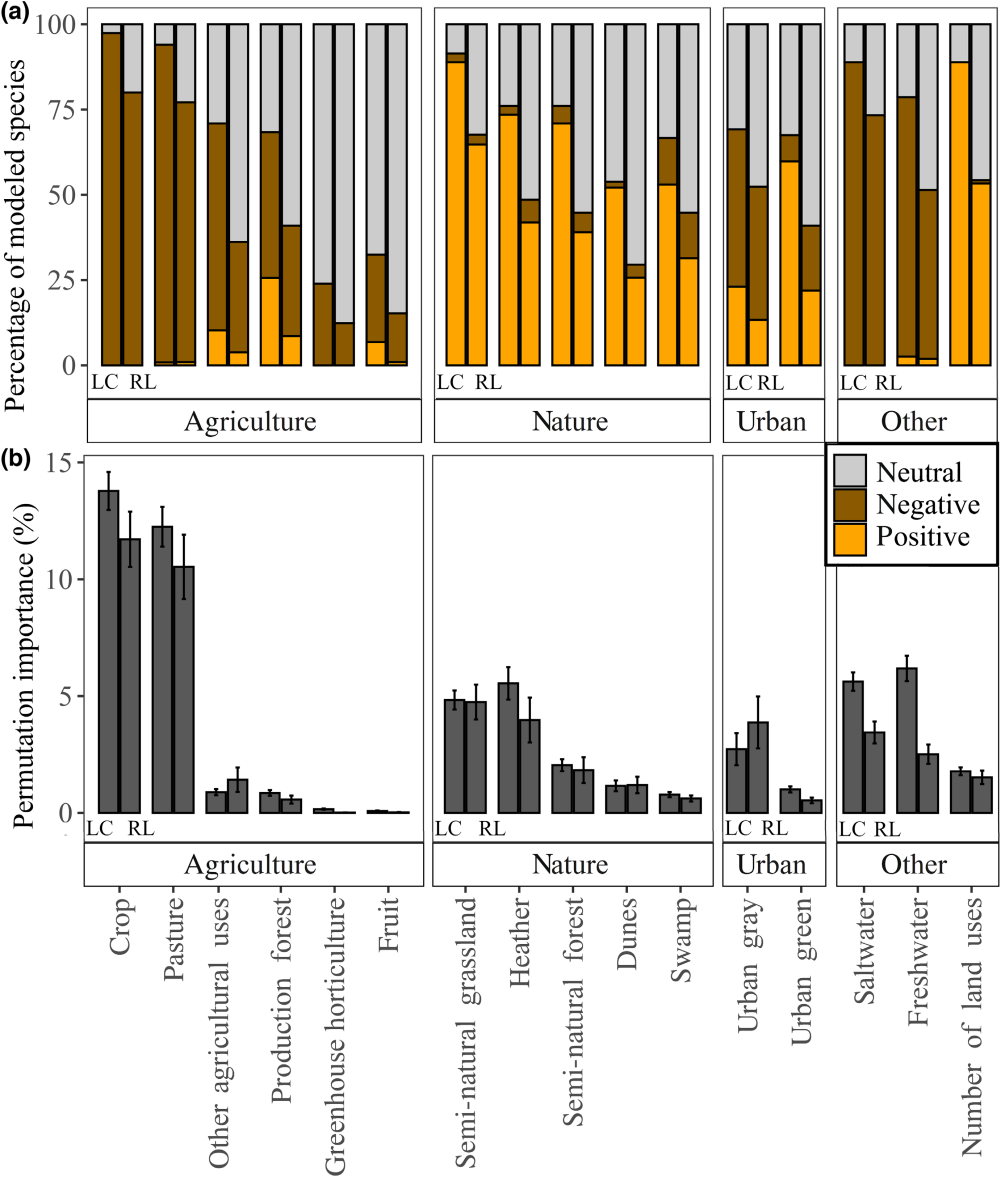
The response to different land use variables in the species distribution models of Least Concern and Red List wild bees (a) and the permutation importance with the standard error (b). The bars represent the percentage of species that showed a negative correlation (dark orange), a positive correlation (light orange), or no significant correlation (neutral) with the land use variable. For every land use category, the left stacked bars represent the Least Concern species, and the right stacked bars represent the Red List species.

When the individual variables were compared, the LC wild bees had a significantly higher permutation importance (Kruskal–Wallis chi‐squared [*χ* ^2^] = 1233.3) than the RL species for number of land uses (*p* = .0016), crop (*p* = .0038), freshwater (*p* < .001), saltwater (*p* < .001), heather (*p* < .001), production forest (*p* = .0096), semi‐natural forest (*p* = .0033), semi‐natural grassland (*p* < .001), and urban green (*p* = .031), while the LC species had a lower permutation importance for pasture (*p* < .001). Crop and pasture were the variables with the average highest permutation importance (12.80 ± 0.71; 11.44 ± 0.79, respectively; Figure 5b).

**FIGURE 6:**
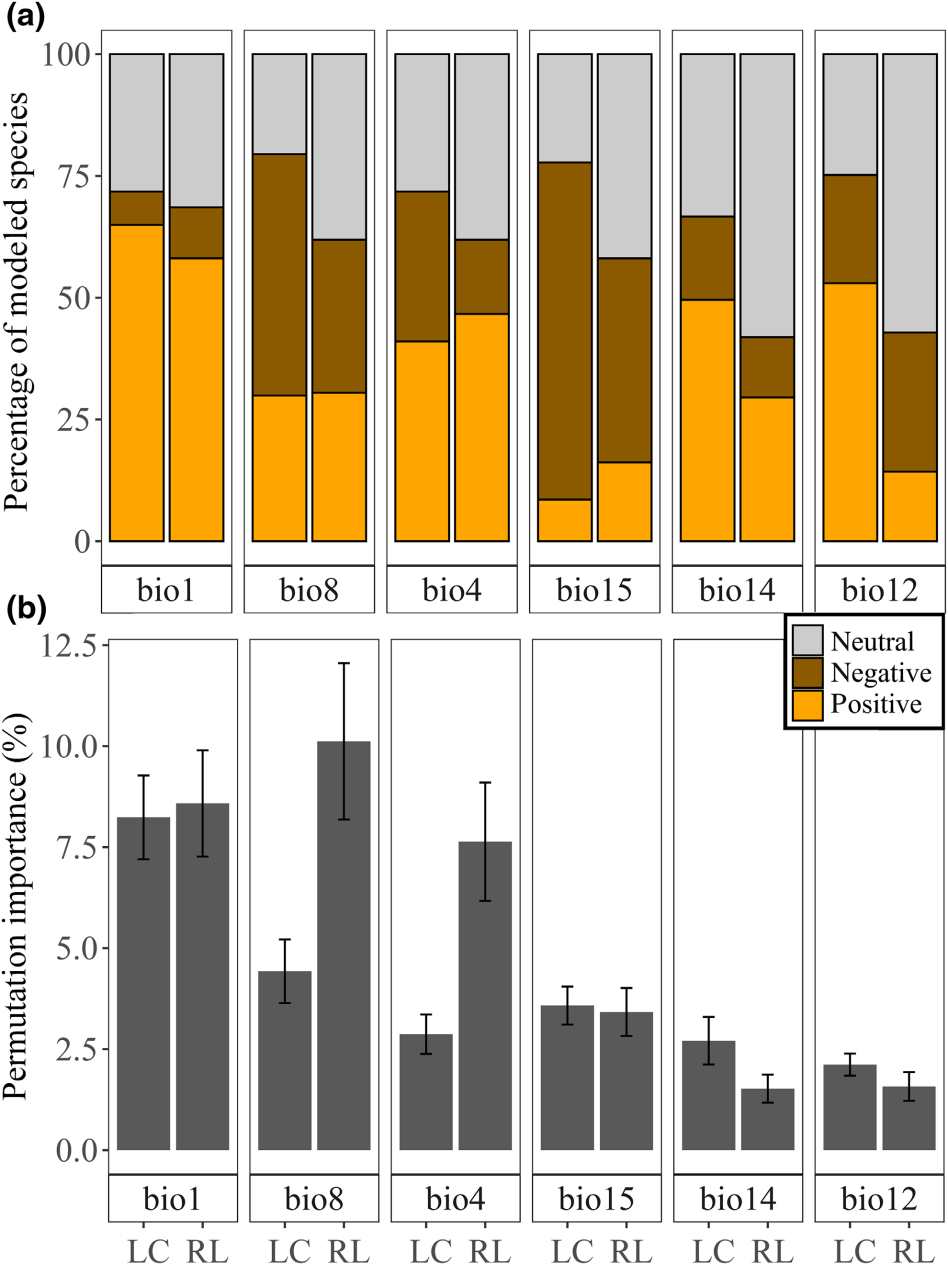
The response to annual mean temperature (bio 1), temperature seasonality (bio 4), mean daily mean air temperatures of the wettest quarter (bio 8), annual precipitation amount (bio 12), precipitation amount of the driest month (bio 14) and precipitation seasonality (bio 15) in the species distribution models of Least Concern and Red List Dutch wild bees (a) and the permutation importance with the standard error (b). The bars represent the percentage of species that showed a negative correlation (dark orange), or a positive correlation (light orange) or no significant correlation (neutral) with the bioclimatic variable.

The LC wild bees had a significantly higher permutation importance than RL wild bees for bioclim 12 (2.12 ± 0.27 and 1.58 ± 0.36; *χ* ^2^ = 121.39; *p* = .0019), bioclim 14 (2.12 ± 0.27 and 1.58 ± 0.36; *χ* ^2^ = 121.39; *p* = .0019) and bioclim 15 (2.12 ± 0.27 and 1.58 ± 0.36; Kruskal–Wallis chi‐squared [*χ* ^2^] = 121.39; *p* = .0019; Figure 6b).

Comparing the individual soil variables, the LC species had a significantly higher permutation importance (*χ* ^2^ = 275.56) for light clay (*p* = .011), light zavel (*p* = .0047) and loam (*p* < .001), and lower for peat (*p* < .001). For all wild bees, sand (7.82 ± 0.91) and peat (3.73 ± 0.33) had a higher permutation importance than the other variables (Figure S1B).

### 3.3 Response toward land use, climate, and soil

The LC and RL bees showed almost exclusively negative responses or no response to cropland and pasture and this effect was especially strong for species with larger extent of occurrence (Figure 5a; Figure S2A). For the majority of land use, climate, and soil variables LC species more often showed a positive or negative response compared to RL species for most land use variables (Appendix S1). When the ratio between positive response and negative response was compared between LC and RL species (*χ* ^2^ = 24.27; *p* < .001), LC species showed more often a positive response to urban green (*p* < .001).

In general, LC species had significantly more often positive responses to multiple natural land use variables compared to RL species (Kruskal–Wallis chi‐squared [*χ* ^2^] = 254.74; *p* < .001; Figure S3).

Comparing the positive and negative ratio for LC and RL species in their response toward climate variables, LC species showed more often a positive response, as opposed to a negative response, (*χ* ^2^ = 0.14; *p* < .001) for bioclim 12 (*p* < .001) and more often a negative response for bioclim 4 (*p* = .049) and bioclim 15 (*p* = .029; Figure 6a). All wild bee species had consistently positive responses to mean annual air temperature (bioclim 1), temperature seasonality (bioclim 4), and precipitation amount of the driest month (bioclim 14), also across different extent of occurrences (Figure S2B). The bees had mainly negative responses to precipitation seasonality (bioclim 15).

For the soil variables, the ratio between positive and negative response was not significantly different between LC and RL species. The majority of the modeled species showed a positive response toward peat, light clay, and heavy clay, while both negative and positive responses were found for sand, light zavel, and loam (Figure S1A).

### 3.4 Difference between European and Dutch red list

All 222 modeled species had a Dutch Red List status, but 19.37% did not have a European Red List status and were labeled as data deficient (DD), indicating missing distribution or abundance information for large parts of their European range (Nieto et al., 2014). The Red List status of species occurring on both lists was as follows: 93 species with a LC status on both lists, 13 species with a RL status on both lists, 67 species with a RL status in the Netherlands and LC status in Europe, and 6 species with a LC status in the Netherlands and a RL status in Europe. When we performed the analyses using the conservation status of the European Red List we found the same trend as for the Dutch Red List with a higher permutation importance of land use for the LC species compared to the RL species (59.05% ± 1.82% and 38.30% ± 4.64%) and a lower permutation importance of climate (25.37% ± 1.65% and 40.41% ± 5.91%). However, with the limited number of RL species on the European Red List (19) these differences were not significant. The DD species were more similar to the RL species: 44.38% ± 4.08% summed permutation importance of land use and 33.11% ± 3.53% contribution of climate. The land use summed permutation importance was significantly different (Kruskal–Wallis chi‐squared [*χ* ^2^] = 244.9; *p* < .001) from the LC species (*p* = .029). Differences in the extent of occurrence showed a similar trend, when the European Red List was used, and the DD species had a similar average to the RL species. The extent of occurrence was different (*χ* ^2^ = 55.09; *p* < .001) between LC and RL species (*p* = .0033) and LC and DD species (*p* = .035).

## 4 DISCUSSION

Through an analysis of variable contribution of climate, land use, and soil, we have presented differences in the factors driving the distribution of not‐threatened (LC) and threatened (RL) wild bee species in the Netherlands. Our results demonstrate that climatic variables exerted a greater influence on the models of RL species, while land use variables were more important for the LC species, although this pattern varied based on how widely distributed a species was. Interestingly, this greater importance of climate for RL species was associated with distributions that reflect the climate extremes of the Netherlands. Global climate change may result in a higher mortality of species that are not able to acclimate fast enough to new climatic conditions or a disruption of ecological interactions due to a difference in the capability of thermal acclimation between species (Rohr et al., 2018). In the case of insects, climate can particularly affect those insects that are limited by their climatic envelope through mechanisms such as the induction of torpor or aestivation or disturbance of the diapause (Sands, 2018).

A potential threat to pollinating insects is a decrease in synchrony between the bee and the pollinated flower phenology (Hegland et al., 2009; Pyke et al., 2016). This suggests that specialist species, with a limited climate envelope, would be particularly at risk in climatic change scenarios and it has been observed in other species that specialists are declining and slowly being replaced by generalist species (Clavel et al., 2011). Due to the limited range of these species, they occupy a subset of the Dutch climate and will be more vulnerable to changes in any direction. Moreover, an important factor in the decline of bees is their host pollen plant (Scheper et al., 2014). The difference in climate contribution between RL and LC species may also originate from populations living at the upper or lower limits of their potential environmental niche. Other research has shown that endangered plants tend to live in the environmental extremes, such as very moist or dry areas (Boulangeat et al., 2012; Van Bodegom et al., 2014) and we found a similar trend for bees. Several RL bee species are found only in the South of the Netherlands as climatic conditions are more suitable there (Peeters et al., 2012). The European Red List assessment is not restrained by the smaller geographic region of the Netherlands. Using the categories of the European Red List, we still found a higher contribution of climate for the RL species and a higher contribution of land use for the LC species. These results support that RL species are more affected by climate, even when potential niche truncation due to restricting the focal extent to political boundaries, is considered.

The higher contribution of land use variables in the models of the LC species and more widespread distribution suggests that the distribution of LC species is mainly affected by land use variables that vary on a small scale in contrast to climate variables that are known to act on a broader scale (Santos et al., 2021). After accounting for the effect of the extent of occurrence on land use variables, the trend was the opposite, and the RL species had a higher importance of land use to their models. These results could indicate a higher negative effect of land use categories, such as pasture and crops. Surprisingly, we found no negative responses to the number of land use classes, although the RL species had fewer positive responses. Although the number of land uses per grid cells has its limitations, for example, it does not take into account the proportion of each land use category similar to Oehri et al. (2020), it can give an indication of landscape heterogeneity. A more heterogeneous landscape could provide nesting opportunities and/or suitable habitats for more species of pollinators than homogeneous landscapes (Andersson et al., 2014; Bennett & Lovell, 2019). In general, an increase in land uses can have positive effects such as landscape complementation, habitat diversity, and spreading of risk (Fahrig et al., 2019). However, very high numbers of land uses in a landscape could indicate a fragmented landscape, with a lack of refuge for the pollinators in homogeneous landscapes with only agriculture or urban area (Jauker et al., 2009; Kleijn et al., 2011). The less positive responses to the number of land uses by the RL species could indicate a higher sensitivity to habitat fragmentation.

Our study, consistent with research on bumblebees in Belgium (Marshall et al., 2021) revealed mixed responses of wild bees to urban habitats. The higher number of positive responses to urban habitats for LC species, compared to RL species may reflect specific traits that are conducive to city dwelling. Previous studies have suggested that urban areas tend to host smaller bees that are cavity‐nesting and have later activity periods and eusocial behavior (Banaszak‐Cibicka & Żmihorski, 2012; Buchholz & Egerer, 2020). In contrast, bees showed no positive response to crops and pastures, which constitute a sizable portion of the Dutch landscape. Studies have found more often a lower species richness in agricultural areas compared to urban and natural areas (reviewed in Prendergast et al., 2022). The exclusively negative responses to crops and pasture in this study could be due to the intensively managed agriculture in the Netherlands (Scheper et al., 2014) and the exclusion of landscape elements, which can provide essential nesting and floral resources for pollinators (Garibaldi et al., 2014; Timberlake et al., 2019). Notably, RL were observed more often in natural areas than LC bees, but they also demonstrated a greater selectivity toward certain natural land use types. The increased natural land use selectivity for RL bees is supported by the lower average number of favorable natural land use types that they responded positively toward. These results support earlier reports suggesting that the niche of RL species is narrower than that of LC species (Pandolfo et al., 2016; Pärtel et al., 2004; Richards et al., 2021). The more specific natural land use requirements for the RL species would be in line with a study on plant species in the Netherlands that found a preference of RL species toward locations with a higher natural land cover (Pan et al., 2021). New conservation strategies have emerged, and these strategies focus more on the RL species and their habitats (Volis, 2019) and propose to examine the physiological and ecological needs of the RL species and identify locations for assisted colonization (Tomlinson et al., 2022). Our findings highlight the need to preserve specific natural land uses, to safeguard the survival of threatened RL species. These results underscore the importance of incorporating such habitats into existing protected areas and conservation strategies, with a species‐specific approach (Ghisbain et al., 2020). Our analyses show that nearly all the modeled bees exhibited a negative response to agricultural areas, indicating that improvements to agriculture may offer some relief to LC bees, but are unlikely to benefit RL species. Even though landscape elements can provide a habitat even for threatened bees (Marja et al., 2018), it is unlikely that small strips of farmland can replace the vast expanses of flower‐rich hay meadows and other extensive agricultural habitats that once dominated the landscape.

Even though researchers have proposed that models can provide valuable information for the Red List assessments, it has rarely been implemented (reviewed in Cazalis et al., 2022). This knowledge‐implementation gap remains and efforts could be made to improve collaboration between Red List practitioners and academic researchers (Cazalis et al., 2022). Achieving such collaboration, could allow models, such as the models used in this study, to significantly contribute to the Red List assessments. A recent study applied a machine‐learning technique to the data deficient species of the Red List and results suggested that these species are more threatened than the data sufficient species (Borgelt et al., 2022). Our study supports that the wild bees listed as threatened on the European Red List were more similar to the Data deficient species than the non‐threatened bees in their response to land use and climate and their extent of occurrence.

The discrepancies in the importance of land use and climate on the distribution of LC and RL species highlight the need for further investigation into the underlying mechanisms behind this difference. Such research can enhance our understanding of species‐specific factors that increase the risk of becoming threatened. Moreover, identifying how climate and land use interact and impact population dynamics (Schulte To Bühne et al., 2021) is crucial for predicting the future distribution of LC and RL species, which are likely to respond differently to future change (Marshall et al., 2018).

## 5 CONCLUSIONS

In this study we compared the contribution of land use, climate and soil to the species distribution models of wild bees in the Netherlands. Non‐threatened (LC) bees had a higher variable importance of the land use variables to their models, as opposed to the climate variables for the threatened (RL) bees. The threatened bees had a smaller extent of occurrence and occupied areas with more extreme climatic conditions. Additionally, the LC and RL bees differed in their responses toward urban green and selectivity toward natural land uses, and climate variables, such as annual precipitation, annual mean temperature, and precipitation seasonality. Crops and pastures were the main contributing land use variables having a negative correlation with the distribution of nearly all bee species. Surprisingly, bees showed a positive response toward the number of land uses, although the number of RL bees that showed a positive response, was lower. This supports the benefit of a diverse landscape for wild bees or could be a result of the detrimental effect of the omnipresent agricultural area in the Netherlands. The knowledge from this study supports the implementation of appropriate, species‐specific conservation measures, including the preservation of natural areas, and the improvement of land use practices in agricultural and urban areas, which may help mitigate the negative impacts of future global change on species' distributions.

## AUTHOR CONTRIBUTIONS

**Merijn Moens:** Conceptualization (equal); formal analysis (lead); investigation (lead); methodology (lead); validation (lead); visualization (lead); writing – original draft (lead). **Jacobus C. Biesmeijer:** Conceptualization (equal); supervision (equal); writing – review and editing (equal). **Saskia G. T. Klumpers:** Conceptualization (equal); supervision (equal); writing – review and editing (equal). **Leon Marshall:** Conceptualization (equal); supervision (lead); writing – review and editing (lead).

## Supporting information


Data S1.

Data S2.

Appendix S1.

## Data Availability

Script and data have been made publicly available and can be accessed in the Supporting Information.
