# Supplementary material for: Are threatened species special? An assessment of Dutch bees in relation to land use and climate
Source: Ecol Evol. 2023 Jul 26;13(7):e10326. doi: 10.1002/ece3.10326 (PMC10369158; doi:10.1002/ece3.10326)
Supplement: Supplementary file 3 — Appendix S1. [file ECE3-13-e10326-s002.docx]

**Supplementary Figures:**


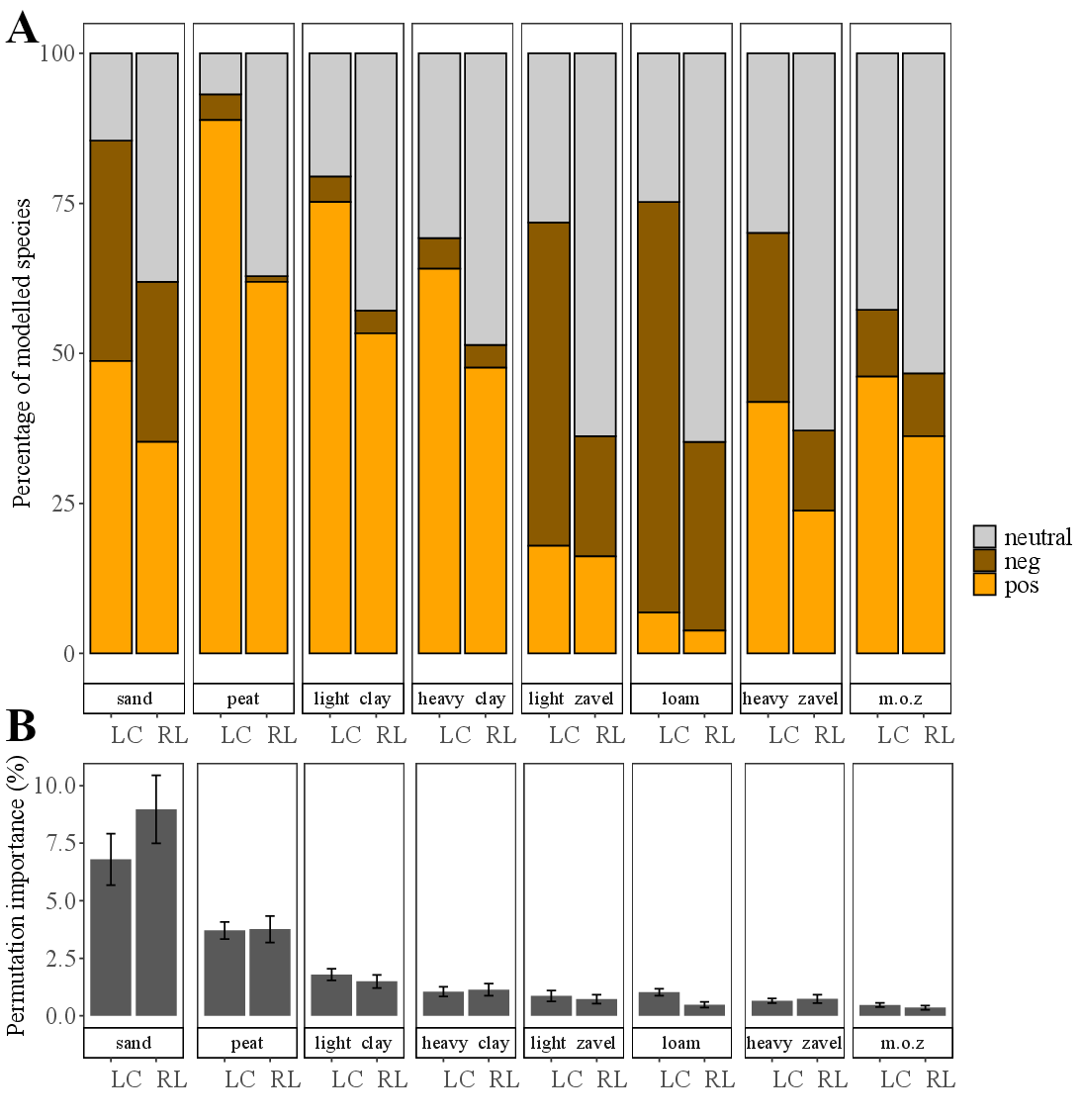


**Supplementary Figure 1:** the response to different soil variables in the species distribution models of Least Concern and Red List Dutch wild bees (A) and the permutation importance with the standard error (B). The bars represent the percentage of species that showed a negative correlation (dark orange), or a positive correlation (light orange) or no significant correlation (neutral) with the soil variable.


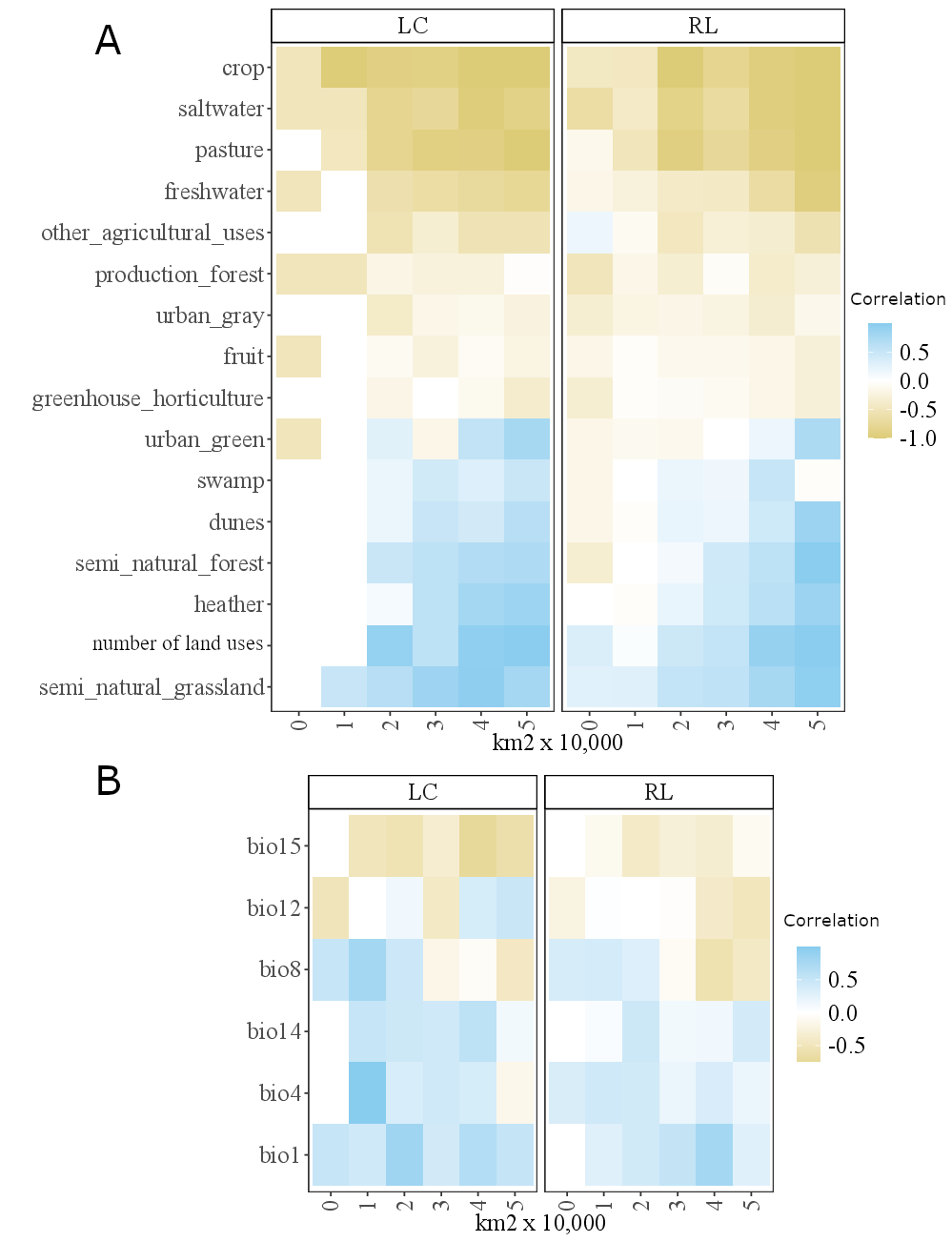


**Supplementary Figure 2:** the response to different land use (A) and climate variables (B) in the species distribution models of Least Concern and Red List Dutch wild bees (A) divided by extent of occurrence (Syfert et al. 2014). The extent of occurrence was aggregated by rounding the extent of occurrence towards the nearest 10,000 km2 group (e.g.: 5200 km2 is rounded towards 10,000 km2 ).


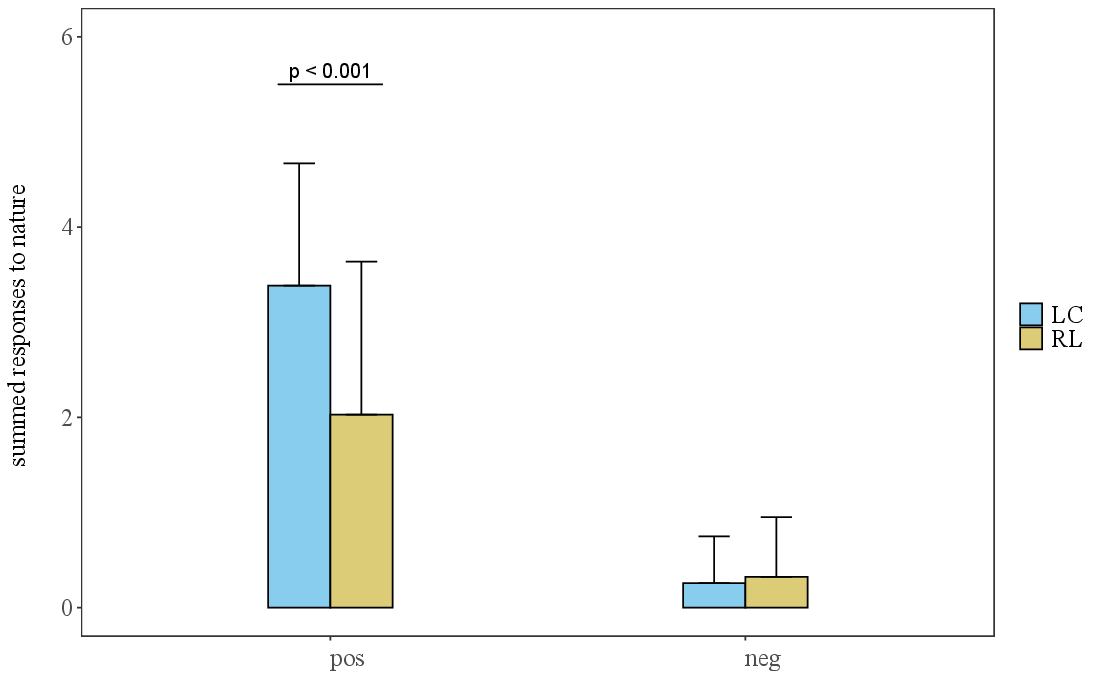


**Supplementary Figure 3:** the average number of positive and negative responses to different natural land use variables for Least Concern (LC) and Red List (RL) Dutch wild bees. The error bars represent the standard deviation.

**Supplementary Tables:**

**Supplementary Table 1:** description of the main soil types in the Netherlands and their characteristics (Silvis and Voskuilen 2016).

| Dutch term | Veen | Moerig op zand | Zand | Leem | Lichte zavel | Zware zavel | Lichte klei | Zware klei |
| --- | --- | --- | --- | --- | --- | --- | --- | --- |
| **English term used** | **Peat** | **m.o.z.** | **Sand** | **Loam** | **Light Zavel** | **Heavy zavel** | **Light clay** | **Heavy clay** |
| **Clay (%)** |  |  |  |  | 8-17.5 | 17.5-25 | 25-35 | 35-45 |
| **Silt (%)** |  |  | < 50 | > 50 |  |  |  |  |
| **Sand (%)** |  |  | > 50 | < 50 |  |  |  |  |
| **Organic matter (%)** | > 15/30 | * | < 15 | < 15 | < 15 | < 18 | < 20 | < 22.5 |

* The upper soil type is less than 40 cm and consists of high organic content.

**Supplementary Table 2:** list of bee species in this study with a Least Concern status or any other status.

| Least concern bee species | Red list bee species |
| --- | --- |
| *Andrena angustior*  *Andrena barbilabris*  *Andrena bicolor*  *Andrena carantonica*  *Andrena chrysosceles*  *Andrena cineraria*  *Andrena clarkella*  *Andrena dorsata*  *Andrena flavipes*  *Andrena florea*  *Andrena fucata*  *Andrena fulva*  *Andrena haemorrhoa*  *Andrena helvola*  *Andrena labiata*  *Andrena lapponica*  *Andrena minutula*  *Andrena minutuloides*  *Andrena mitis*  *Andrena nigroaenea*  *Andrena nitida*  *Andrena praecox*  *Andrena proxima*  *Andrena subopaca*  *Andrena synadelpha*  *Andrena vaga*  *Andrena ventralis*  *Anthidiellum strigatum*  *Anthidium manicatum*  *Anthophora plumipes*  *Bombus campestris*  *Bombus cryptarum*  *Bombus hortorum*  *Bombus hypnorum*  *Bombus lapidarius*  *Bombus lucorum*  *Bombus norvegicus*  *Bombus pascuorum*  *Bombus pratorum*  *Bombus sylvestris*  *Bombus terrestris*  *Ceratina cyanea*  *Chelostoma rapunculi*  *Colletes cunicularius*  *Colletes daviesanus*  *Colletes fodiens*  *Colletes halophilus*  *Colletes hederae*  *Colletes marginatus*  *Colletes similis*  *Colletes succinctus*  *Dasypoda hirtipes*  *Epeoloides coecutiens*  *Epeolus cruciger*  *Epeolus tarsalis*  *Epeolus variegatus*  *Halictus confusus*  *Halictus rubicundus*  *Halictus tumulorum*  *Heriades truncorum*  *Hoplitis leucomelana*  *Hylaeus brevicornis*  *Hylaeus communis*  *Hylaeus confusus*  *Hylaeus cornutus*  *Hylaeus dilatatus*  *Hylaeus gredleri*  *Hylaeus hyalinatus*  *Hylaeus signatus*  *Lasioglossum albipes*  *Lasioglossum calceatum*  *Lasioglossum fratellum*  *Lasioglossum fulvicorne*  *Lasioglossum laticeps*  *Lasioglossum leucopus*  *Lasioglossum leucozonium*  *Lasioglossum lucidulum*  *Lasioglossum minutissimum*  *Lasioglossum morio*  *Lasioglossum pauxillum*  *Lasioglossum punctatissimum*  *Lasioglossum sabulosum*  *Lasioglossum semilucens*  *Lasioglossum sexstrigatum*  *Lasioglossum villosulum*  *Lasioglossum zonulum*  *Macropis europaea*  *Megachile versicolor*  *Megachile willughbiella*  *Melitta haemorrhoidalis*  *Melitta nigricans*  *Nomada alboguttata*  *Nomada conjungens*  *Nomada fabriciana*  *Nomada flava*  *Nomada flavoguttata*  *Nomada fucata*  *Nomada marshamella*  *Nomada panzeri*  *Nomada ruficornis*  *Nomada sheppardana*  *Nomada signata*  *Nomada succincta*  *Osmia bicornis*  *Osmia uncinata*  *Panurgus calcaratus*  *Sphecodes albilabris*  *Sphecodes crassus*  *Sphecodes geoffrellus*  *Sphecodes gibbus*  *Sphecodes longulus*  *Sphecodes marginatus*  *Sphecodes miniatus*  *Sphecodes monilicornis*  *Sphecodes pellucidus*  *Sphecodes puncticeps*  *Sphecodes reticulatus* | *Andrena apicata*  *Andrena argentata*  *Andrena bimaculata*  *Andrena denticulata*  *Andrena fulvago*  *Andrena fulvida*  *Andrena fuscipes*  *Andrena gravida*  *Andrena hattorfiana*  *Andrena humilis*  *Andrena labialis*  *Andrena lathyri*  *Andrena nigriceps*  *Andrena ovatula*  *Andrena pilipes*  *Andrena rosae*  *Andrena ruficrus*  *Andrena semilaevis*  *Andrena tibialis*  *Andrena varians*  *Andrena viridescens*  *Andrena wilkella*  *Anthidium punctatum*  *Anthophora furcata*  *Anthophora quadrimaculata*  *Anthophora retusa*  *Bombus bohemicus*  *Bombus humilis*  *Bombus jonellus*  *Bombus magnus*  *Bombus muscorum*  *Bombus ruderarius*  *Bombus rupestris*  *Bombus vestalis*  *Bombus veteranus*  *Chelostoma campanularum*  *Chelostoma florisomne*  *Coelioxys elongata*  *Coelioxys inermis*  *Coelioxys mandibularis*  *Eucera longicornis*  *Eucera nigrescens*  *Halictus scabiosae*  *Hoplitis adunca*  *Hoplitis claviventris*  *Hylaeus incongruus*  *Hylaeus pectoralis*  *Hylaeus pictipes*  *Hylaeus punctulatissimus*  *Hylaeus rinki*  *Lasioglossum brevicorne*  *Lasioglossum lativentre*  *Lasioglossum malachurum*  *Lasioglossum nitidiusculum*  *Lasioglossum nitidulum*  *Lasioglossum pallens*  *Lasioglossum parvulum*  *Lasioglossum prasinum*  *Lasioglossum quadrinotatulum*  *Lasioglossum quadrinotatum*  *Lasioglossum sexnotatum*  *Lasioglossum xanthopus*  *Macropis fulvipes*  *Megachile centuncularis*  *Megachile circumcincta*  *Megachile lapponica*  *Megachile leachella*  *Megachile ligniseca*  *Megachile maritima*  *Melecta albifrons*  *Melitta leporina*  *Melitta tricincta*  *Nomada armata*  *Nomada bifasciata*  *Nomada femoralis*  *Nomada ferruginata*  *Nomada flavopicta*  *Nomada fulvicornis*  *Nomada fuscicornis*  *Nomada goodeniana*  *Nomada lathburiana*  *Nomada leucophthalma*  *Nomada obscura*  *Nomada rufipes*  *Nomada similis*  *Nomada stigma*  *Nomada striata*  *Nomada zonata*  *Osmia aurulenta*  *Osmia caerulescens*  *Osmia cornuta*  *Osmia leaiana*  *Osmia niveata*  *Osmia spinulosa*  *Panurgus banksianus*  *Sphecodes ephippius*  *Sphecodes ferruginatus*  *Sphecodes rubicundus*  *Sphecodes scabricollis*  *Stelis breviuscula*  *Stelis ornatula*  *Stelis phaeoptera*  *Stelis punctulatissima*  *Stelis signata*  *Xylocopa violacea* |

**Supplementary Table 3:** a standardized graph for reporting species distribution models in (Zurell et al. 2020).

| **ODMAP section** | **ODMAP subsection** | **ODMAP elements** |
| --- | --- | --- |
| Overview | Authorship | **Authors:** M. Moens; K. J. C. Biesmeijer; S. Klumpers; L. Marshall |
|  |  | **Contact email:** merijn.moens@naturalis.nl |
|  |  | **Title:** Are threatened species special: an assessment of Dutch bees in relation to climate and land use |
|  |  | · DOI |
|  | Model objective | **SDM objective/purpose:** ecological inference/ explanation |
|  |  | **Main target output:** permutation importance and response curves |
|  | Taxon | **Taxon:** bees (epifamily Anthophila) |
|  | Location | **Location of study area:** The Netherlands |
|  | Scale of analysis | **Spatial Extent (Lon / Lat):** Longitude 3.35° E – 7.24° E, Latitude 50.75° S – 53.52° S |
|  |  | **Spatial resolution:** 100 m |
|  |  | **Temporal extent/time period:** 2004-2019 |
|  |  | **Type of extent boundary:** administrative (country) |
|  | Biodiversity data overview | **Observation type:** collection of standardised monitoring data, field survey and citizen science. |
|  |  | **Response/data type:** presence only |
|  | Type of predictors | Soil, climate and land use variables. |
|  | Conceptual model | Hypotheses about species-environment relationships: are threatened and non-threatened species affected differently by land use and climate? |
|  | Assumptions | We assume that the sampling is homogeneous across the sampled sites in the Netherlands. Additionally, we assume that the modeled response to the environment represents the species' response to the environment. |
|  | SDM algorithms | **Model algorithms:** MaxEnt |
|  |  | **Justification of model complexity:** feature classes include linear (L), quadratic (Q), hinge (H), threshold (T) and product (P) features. The regularization multiplier ranged from 1 to 15 and the set of feature types were tested in the following combinations: L, L+Q, H, L+Q+H, L+Q + H+P, L+Q+H+P+T. |
|  |  | **Is model averaging/ensemble modelling used?** For every modelled species, model selection is based on lowest AICc.(Burnham & Anderson 2002) |
|  | Model workflow | We modelled 222 bee species. The permutation importance was calculated and the response to a variable was categorized based on the response curve. Linear and non-parametric regression was applied to the results and the results were compared between Least Concern and Red List species. |
|  | Software, codes and data | **Modelling platform:** R (version 3.4.1) with packages dismo version 1.3-3 (Hijmans et al. 2020) and ENMeval version 0.3.1 (Muscarella et al. 2014). |
|  |  | R code in supplementary materials. |
|  |  | Data available in supplementary materials |
| Data | Biodiversity data | **Taxon:** bees (epifamily Anthophila) |
|  |  | **Ecological level:** species and community (individual species are modelled, but analysis is performed on a large number of bee species) |
|  |  | **Biodiversity data source:** observation data from EIS (www.eis-nederland.nl) |
|  |  | **Sampling design**: random |
|  |  | **Sample size per taxon:** from 15 to 4616 observations. |
|  |  | **Country/region:** the Netherlands |
|  |  | **Details on absence data collection:** no absence data |
|  |  | **Details on potential errors and biases in data:** the database consist of data from different sources with potential errors of misidentification or erroneous geo-referenced data. Data may have a sampling bias towards areas that are generally sampled more than other areas. |
|  | Data partitioning | **Selection of training data:** spatial block validation (partitioned geographically; Muscarella et al. 2014) |
|  |  | **Selection of validation data**: (see training data) |
|  | Predictor variables | **State predictor variables used:** climate (6 variables), land use (16 variables) and soil (8 variables). |
|  |  | **Details on data sources:** see paper for explanation data sources. |
|  |  | **Map projection:** EPSG:28992 |
|  |  | **Details on data processing and on spatial, temporal and thematic scaling:** bioclimatic variables were selected based on a correlation threshold of Spearman’s rho = 0.7. |
|  | Transfer data for projection | The focus of this research is on the permutation importance and the response curves; not the projection. |
|  | Multicollinearity | Variables were tested for collinearity with a spearman's correlation. None of the variables had a correlation coefficient higher than 0.7. |
|  | Model settings | **Models settings:** Model settings depend on model selection (see *“SDM algorithms*”) |
|  | Model estimates | **Assessment of model coefficients:** -- |
|  |  | **Assessment of variable importance:** the permutation importance was analyzed between a large number of modelled species (222 species). |
| Assessment | Performance statistics | **Performance statistics:** The corrected Aikake Information Criteria (AICc).. |
|  |  | **Performance statistics on validation data:** The corrected Aikake Information Criteria (AICc). Other evaluation measures such as the evaluation Area Under the Curve (AUC) of the ROC, the evaluation measure of the partial ROC (Cobos et al. 2019) and the -Continuous Boyce index (CBI; Hirzel et al. 2006) are available in the data in the supplementary materials. |
|  | Plausibility check | Checking the response plots with wild bee experts. |

**Supplementary Table 4:** table showing the output from a linear regression and a non-parametric regression model (Kloke and McKean 2012) for the summed permutation importance of the land use variables.

|  | Parametric | | | | | Non-parametric | | |
| --- | --- | --- | --- | --- | --- | --- | --- | --- |
| Formula | AICc | Fstat | df | R2 | p | Fstat | R2 | p |
| value ~ status+distribution_area | 1951.35 | 69.07 | 219 | 0.38 | 5.5e-24 | 56.95 | 0.34 | 0 |
| value ~ status + distribution_area + status:distribution_area | 1952.25 | 46.47 | 218 | 0.38 | 2.9e-23 | 38.71 | 0.35 | 0 |
| value ~ distribution_area | 1953.91 | 131.37 | 220 | 0.37 | 3.7e-24 | 107.45 | 0.33 | 0 |
| value ~ status | 2046.14 | 11.92 | 220 | 0.047 | 0.00067 | 10.23 | 0.044 | 0.0016 |

**Supplementary Table 5:** table showing the output from a linear regression and a non-parametric regression model (Kloke and McKean 2012) for the summed permutation importance of the climate variables.

|  | Parametric | | | | | Non-parametric | | |
| --- | --- | --- | --- | --- | --- | --- | --- | --- |
| Formula | AICc | Fstat | df | R2 | p | Fstat | R2 | p |
| value ~ status+distribution_area | 1923.14 | 54.63 | 219 | 0.33 | 5.6e-20 | 48.4 | 0.31 | 0 |
| value ~ status + distribution_area + status:distribution_area | 1923.95 | 36.89 | 218 | 0.33 | 2.5e-19 | 33.63 | 0.32 | 0 |
| value ~ distribution_area | 1925.43 | 103.35 | 220 | 0.32 | 3.8e-20 | 82.32 | 0.27 | 0 |
| value ~ status | 2001.75 | 9.28 | 220 | 0.036 | 0.0026 | 6.34 | 0.028 | 0.013 |

**Supplementary Table 6:** table showing the output from a linear regression and a non-parametric regression model (Kloke and McKean 2012) for the summed permutation importance of the soil variables.

|  | Parametric | | | | | | Non-parametric | | |
| --- | --- | --- | --- | --- | --- | --- | --- | --- | --- |
| Formula | AICc | Fstat | df | R2 | p | | Fstat | R2 | p |
| value ~ status+distribution_area | 1831.25 | 1.32 | 218 | 0.0043 | | 0.27 | 0.36 | 0.0049 | 0.78 |
| value ~ status + distribution_area + status:distribution_area | 1829.37 | 1.88 | 219 | 0.0079 | | 0.16 | 0.51 | 0.0046 | 0.6 |
| value ~ distribution_area | 1827.46 | 3.61 | 220 | 0.012 | | 0.059 | 0.75 | 0.0034 | 0.39 |
| value ~ status | 1830.64 | 0.42 | 220 | -0.0026 | | 0.52 | 0.00077 | 3.5e-06 | 0.98 |

**References:**

Kloke, J. D. and McKean, J. W. 2012. Rfit: rank-based estimation for linear models. - R J. 4: 57.

Silvis, H. and Voskuilen, M. 2016. Grondsoort en grondprijs. in press.

Syfert, M. M. et al. 2014. Using species distribution models to inform IUCN Red List assessments. - Biol Conserv 177: 174–184.

Zurell, D. et al. 2020. A standard protocol for reporting species distribution models. - Ecography 43: 1261–1277.
